# Microvasculature remodeling in the mouse lower gut during inflammaging

**DOI:** 10.1038/srep39848

**Published:** 2017-01-03

**Authors:** Jae-Ho Jeong, KwangSoo Kim, Daejin Lim, Kun-Hee Kim, Hyung-Seok Kim, Sungsu Lee, Joo-Hye Song, Byoung-Gon Moon, Hyon E. Choy, Sang Chul Park

**Affiliations:** 1 Department of Microbiology, Chonnam National University Medical School, Republic of Korea; 2 Department of Molecular Medicine(BK21plus), Chonnam National University Graduate School, Republic of Korea; 3 Department of Forensic Medicine, Chonnam National University Medical School, Republic of Korea; 4 Department of Otolaryngology-Head and Neck Surgery, Chonnam National University Hospital, Gwangju, Republic of Korea; 5 Well Aging Research Center, SAIT. Suwon, Gyeonggido, Republic of Korea; 6 Department of New Biology, Well Aging Research Center, DGIST, Daegu, Republic of Korea

## Abstract

Inflammaging is defined as low-grade, chronic, systemic inflammation in aging, in the absence of overt infection. Age-associated deterioration of gastrointestinal function could be ascribed to the inflammaging, although evidence is yet to emerge. Here we show that microvessels in aging mouse intestine were progressively deprived of supportive structures, microvessel-associated pericytes and adherens junction protein vascular endothelial (VE)-cadherin, and became leaky. This alteration was ascribed to up-regulation of angiopoetin-2 in microvascular endothelial cells. Up-regulation of the angiopoietin-2 was by TNF-α, originated from M2-like residential CD206^+^ macrophages, proportion of which increases as animal ages. It was concluded that antigenic burdens encountered in intestine throughout life create the condition of chronic stage of inflammation, which accumulates M2-like macrophages expressing TNF-α. The TNF-α induces vascular leakage to facilitate recruitment of immune cells into intestine under the chronic inflammatory setting.

Aging is a dynamic process of life-long adaptation of the animal to ever changing internal and external environments, resulting in body function decline. Of many proposed mechanisms underlying aging, inflammaging theory proposes that chronic low-grade inflammatory status caused by life-time exposure of animals to a variety of antigens contributes to age-associated morbidity and mortality^1^. An hallmark of age-associated chronic inflammation would be macrophage infiltration^2^. In intestine, the epithelial lining separates internal organs from the enteric environment loaded with various foreign substances including microbiota and its metabolic products as well as nutrients and wastes^3^,^4^. The lamina propria (LP) lying beneath the enterocytes in the intestinal villi especially that in the lower part, houses a largest pool of macrophages for maintaining mucosal homeostasis against the gut microbiota and for the constant need of epithelial renewal^5^. Age-associated deterioration of gastrointestinal function^6^,^7^ could be ascribed to the inflammaging, although substantial evidence is yet to emerge.

The LP contains microvessels as well as a central lacteal (lymph vessel) and lymphoid tissue, in addition to immune cells. Integrity of the microvessel network is critical in maintaining its robustness. The angiopoietin–TIE 1/2 receptor signaling is a vascular-specific receptor tyrosine kinase pathway that is essential for assembly and maintenance of the microvessel network^8^. TIE-2 is differentially regulated by two ligands, angiopoietin 1 (ANG1), an activating agonist of the pathway expressed predominantly in perivascular cells, and angiopoietin 2 (ANG2), a corresponding antagonist produced by microvascular endothelial cells. ANG1 signaling is critical in the stabilization and maturation of vessels by recruiting mural cells, while ANG2 antagonizes the effect of ANG1–mediated stimulation of TIE-2 under the condition of chronic inflammation leading to vessel destabilization and pericyte dropout. In addition, TIE-2 signaling mediated by ANG1/2 is known to be involved in controlling endothelial cell^9^ permeability^8^. The ANG2 binding to TIE-2 triggers degradation of an adherens junction protein vascular endothelial (VE)-cadherin in the junctional complexes via non-receptor Tyr kinase Src, thereby increasing EC permeability. It has been shown that ANG2 expression is up-regulated in many inflammatory and angiogenic settings by several factors including TNF-α, VEGF-A, and hypoxia^10^,^11^. Age-associated deterioration of gastrointestinal function could be ascribed to the increased EC permeability.

## Results

### ANG2-mediated destabilization of microvessel network in aged animals

**Figure 1:**
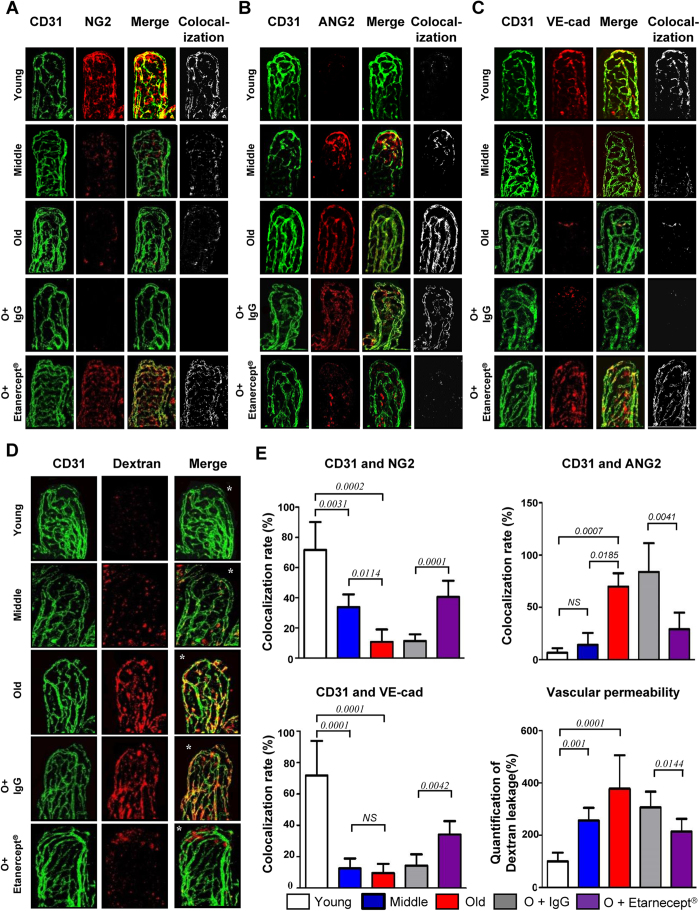
Remodeling of microvasculature in LP of aging mouse lower gut via up-regulation of ANG2. Presence of indicated molecules (**A** to **C**) in lower gut of young, middle aged, and old mice were shown by a whole mount confocal microscopic IF image of LP in using specific antibodies (CD31: vascular endothelial cells, NG2: pericytes, VE-cad: adherens junction protein, ANG2: angiopoietin 2, 400x magnifications) (n = 10 for each groups). O +Etanercept represents old mice treated with Etanercept; O+IgG represents old mice treated with IgG. The right-most columns show merged images produced with LSM image browser software (Carl Zeiss). (**D**) shows leakage of 70-kDa TRITC-dextran from the CD31^+^ vessels in LP of aging mouse gut. High magnification of images at villi tip (*) were shown in Supplementary Fig. 3. (**E**) shows quantification of co-localization indices (the last columns) of (**A**) to (**C**). Quantification of extravascular TRITC-dextran was also included (vascular permeability).

To elucidate the nature of aging-dependent gastrointestinal deterioration, we examined small intestine in the female mice (BL/6) at three different age groups: young (3-month), middle age (12-month), and old age (22-month or older). Networks of CD31^+^ endothelial cells^9^ in LP of the lower part were examined by confocal microscopy after immunofluorescence (IF) staining (Fig. 1). No remarkable difference was noted in the extent of network in the aged mice compared to young and middle age mice. Nor was any sign of vessel growth (sprouting) except the prominent thickening of CD31^+^ vessels in the aging mice. Subsequently, we examined the microvessel-associated pericytes that normally surround vascular EC to support structural stability. Whole mount of the intestines were stained for pericyte-specific Neuron-glial antigen 2 (NG2) marker. Most notably, microvessels in the old mice were found to be virtually free of pericytes (Fig. 1A). This was further verified by another pericyte marker PDFGR-β^12^ which appeared to be virtually depleted in old mice as well (Supplementary Fig. 1).

It has been reported that the loss of pericytes contributes to the pathogenesis of various types of inflammatory disorders^13^. ANG2 signaling is known to antagonize the effect of ANG1–mediated stimulation of TIE-2 under the condition of chronic inflammation leading to vessel destabilization and pericyte dropout by antagonizing the effect of ANG1–mediated stimulation of TIE-2^8^. Thus, the loss of pericyte in aging animals might be ascribed to an alteration of angiopoietin-TIE signaling. We determined the level of ANG2 protein in CD31^+^-endothelial network after IF staining and observed notable age-dependent increase especially from the tip area of EC network (Fig. 1B). ANG2 expression was also determined and found to increase in tissue lysates prepared from the entire intestine as assessed by quantitative PCR (Q-PCR) analyses (Supplementary Fig. 2).

Since ANG2 binding to TIE-2 also triggers degradation of the VE-cadherin, we next examined the expression of VE-Cadherin in CD31^+^ EC in LP after IF staining and observed age-dependent loss (Fig. 1C). We hypothesized that deprivation of VE-cadherin may have destabilized the inter-endothelial junction and thus promotes EC permeability. To test this hypothesis, the EC permeability was assessed using TRITC (tetramethylrhodamine)-conjugated 70-kDa dextran as a tracer. Previous report showed that 70-kDa dextran could readily leak out through EC junctions under inflammatory conditions, but not through normal EC junctions^14^. Consistently, we observed that 70-kDa TRITC-dextran was well-retained in blood of young animal, but leaked into the interstitial space in LP in aged animals: marked leakage was observed within 30 min after intravenous injection (Fig. 1D; Supplementary Fig. 3). Fluorometric analysis of TRITC-dextran leakage in the entire small intestine corroborated the increase in vascular permeability in aged mice (Supplementary Fig. 4). It was conjectured that a constituent of the intestine increased by aging expressed any one or combination of those cytokines known to up-regulate ANG2, TNF-α, VEGF-A, and hypoxia^10^,^11^, that resulted in the leaky vasculature.

### Up-regulation of ANG2 in EC by TNF-α secreted by M2-like macrophages in aging animals

**Figure 2:**
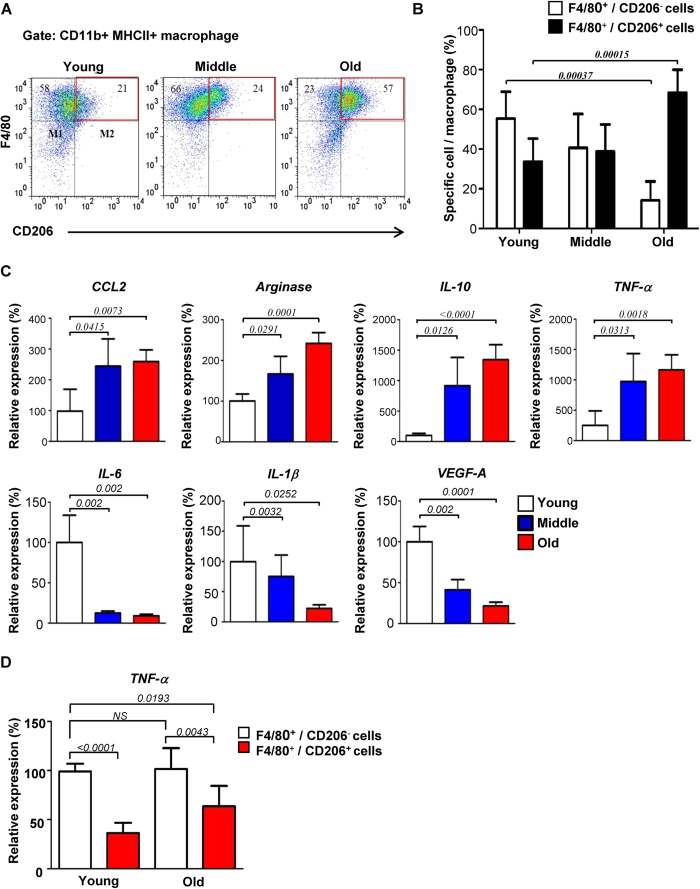
Characterization of macrophages cumulated in LP of aging mouse gut. (**A**) CD11b^+^ MHCII^+^ cells in LP of entire mouse gut from three age groups were sorted into F4/80^+^CD206^+^ and F4/80^+^CD206^−^ macrophages by flow cytometry. This figure shows a representative of five independent experiments. (**B**) shows the quantification. (**C**) Relevant gene expression profile of the F4/80^+^ MHCII^+^ cells. The gene expression levels were determined by Q-PCR. (**D**) TNF-α expression in the F4/80^+^CD206^+^ and F4/80^+^CD206^−^ macrophages of young and old mice guts.

As macrophage infiltration has been proposed to be a hallmark of age-associated chronic inflammation^2^, we reasoned that the source of ANG2 inducing factor may be the macrophages in the LP. Quantification of F4/80^+^ macrophages in LP and also in the entire intestine revealed no age-dependent change (data not shown). The macrophages are broadly categorized into two main subtypes, CD206^-^ pro-inflammatory M1 and CD206^+^ anti-inflammatory M2. M1 macrophages with pro-inflammatory cytokines are important for host defense, but could cause considerable damage to the host. Innate signals such as interleukin 4 (IL-4) are known to differentiate resident macrophages into M2 subtype expressing mannose receptor (CD206)^15^. To determine the distribution of CD206^+^ and CD206^−^ cells among F4/80^+^ macrophages in the LP of entire intestine, we performed fluorescence activated cell sorting (FACS) analysis (Fig. 2A and B). Fraction of the CD206^+^ macrophages increased, while CD206^−^ macrophages concomitantly declined as animal aged: the ratio of CD206^+^ macrophages vs CD206^−^ macrophages was about 3:5 in young mice, but later changed to 4:1 at old age. Analysis of gene expression profile of the F4/80^+^ MHCII^+^ macrophages obtained by FACS revealed increased expression of M2-associated cytokines, CCL2, arginase, and anti-inflammatory IL-10, and decreased expression of M1-associated IL-6 and IL-1β, as animal aged (Fig. 2C)^16^. Notably, TNF-α, a signature of systemic inflammation known to be expressed in M1 subtype, was up-regulated in the F4/80^+^ MHCII^+^ macrophages of the aged mice^17^. This was further verified by determining TNF-α in intestinal lysate and the macrophages isolated from LP of young and old mice (Supplementary Fig. 5). Over 2-fold increased expression was detected in both samples from old mice. To identify the source of TNF-α, CD206^+^ and CD206^−^ cells among F4/80^+^ macrophages from young and old mice were separated and analyzed for TNF-α expression. TNF-α expressions from CD206^+^ cells from old animals was increased about 2-fold compare with that from young animals while that from CD206^−^ cells were more or less the same (Fig. 2D). These results suggested that gut-resident macrophages in aging animal may not readily fit into the typical classification M1-M2 paradigm. Moreover, expression of M1-associated VEGF-A was reduced in the aged animal as well^16^. Taken together, we hypothesized that TNF-α produced by resident macrophages in aging animals would be important for the up-regulation of ANG2 in ECs, resulting in the deprivation of pericytes that in turn elicits leaky vasculature in LP of aged intestinal villi.

**Figure 3:**
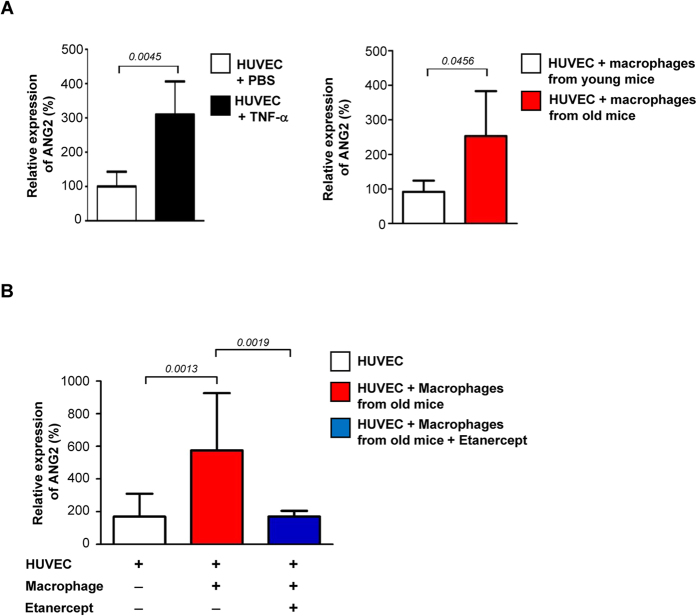
Up-regulation of ANG2 by TNF-α. (**A**) HUVEC grown in EBM2 media (EGM**™-**2 BulletKit™, Lonza) were treated with either TNF-α (Sigma Aldrich, 10 ng/ml) for 10 hrs (left panel) or co-cultured in transwell (Corning, 3 nm pore) with macrophages isolated from young and old mice gut (n = 4) for 48 hrs (right panel). (**B**) HUVEC were co-cultured in transwell with macropahges isolated from old mice gut for 10 hrs in the presence or absence of etanercept (Enbrel;^**®**^ Pfizer, 10 μg/mll). ANG2 expression was determined by Q-PCR.

We further corroborate our hypothesis by determining expression of ANG2 from Human Umbilical Vein Endothelial Cells (HUVEC) treated with TNF-α or co-cultured in transwells with macrophages isolated from the intestines of young or old mice (n=4). TNF-α up-regulated the ANG2 expression by ~3-fold, while co-culturing with macrophages from old mice up-regulated ANG2 by ~2.5-fold compared to that from young mice (Fig. 3A). This up-regulation was nullified by treatment with a specific inhibitor etanercept, a recombinant soluble TNF-α receptor (Fig. 3B)^18^. We then blocked the TNF-α function in old mice using the etanercept. Old animals that received etanercept for 12 days were euthanized and subsequently examined for the presence of pericytes and the expression of ANG2 and VE-cadherin in endothelial network in LP (bottom two rows in Fig. 1A–C). The etanercept treatment resulted in restoration of VE-cadherin and down-regulation of ANG2, consequence of which was reinstatement of pericytes. (Fig. 1E shows quantifications). EC integrity was also determined using 70-kDa TRITC-dextran and found to be restored as well (bottom two rows in Fig. 1D). By comparison, no sign of restoration was observed in the aged mice that received IgG, a negative control. It has been reported that TNF-α initiates vascular remodeling by priming EC for sprouting and promoting pericyte recruitment when secreted by activated macrophages^16^. Under this chronic inflammatory setting, however, TNF-α from resident macrophages is playing an adverse role by inducing ANG2 expression in EC, resulting in increased vascular permeability.

## Discussion

**Figure 4:**
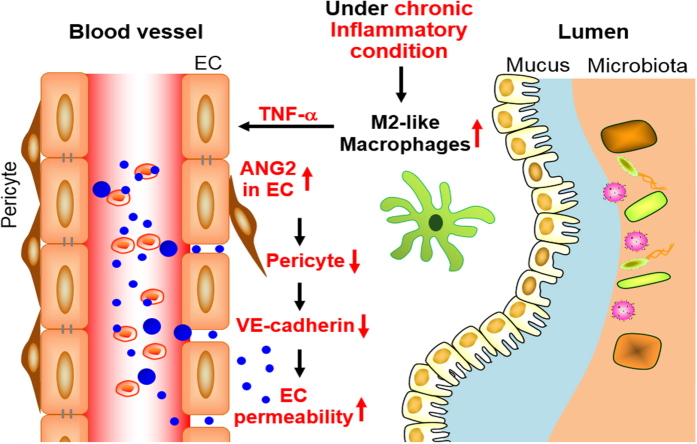
Schematic summary. Under the condition of chronic inflammation, TNF-α-expressing M2-like macrophages are accumulated in LP of aging animal intestine. The TNF-α upregulates ANG2 expression in microvascular endothelial cells that skews the angiopoetin-TIE-2 signaling in neighboring vascular EC to inflammatory settings by removing supportive structures: microvessel-associated pericytes and adherens junction protein vascular endothelial (VE)-cadherin at the cell-cell junction. Consequently, microvessels in aged mice became leaky for recruitment of immune cells.

Homeostatic status of the intestine depends on a complex interplay between age-associated gut microenvironment changes and repopulation of resident immune cells^19^. It has been reported that the total number of F4/80^+^ CD11b^+^ macrophages in the mouse colon is little at birth, but increases progressively to adulthood (5 to 9 weeks) in microbiota-dependent manner^20^. In this study, we propose that the antigenic burden encountered in the intestine causes macrophage infiltration during the first few months after birth and that is sustained throughout life (Fig. 4). Under the condition of chronic inflammation, it stands to reason to polarize macrophage toward the M2-like subtype to avoid tissue injury and eventual chaotic consequences caused by activated M1 macrophage. Consistently, it has been reported that total macrophages and myeloid derived suppressor cells cumulated in the spleens and bone marrow of aged mice were mostly anti-inflammatory M2 cells in aged mice^21^. Macrophages are the sources of both pro-angiogenic and anti-angiogenic factors, which can differentially guide vascular network formation under many pathological conditions. We therefore propose that TNF-α derived from the macrophages in aged animals skews the angiopoetin-TIE-2 signaling in vascular EC to inflammatory settings that would facilitate recruitment of immune cells through ECs. Such increase in vasculature permeability entails modulation of EC network such as loss of VE-cadherin and pericyte^22^, as demonstrated in this study. Together, our study demonstrates for the first time, to the best of our knowledge, that sustained aggravation of inflammation leads to age-related structural changes in organ.

## Materials and Methods

### Mice

Female mice at 3-month, 12-month and 22-month (or older) old aged C57BL/6 J were used for this study. All mice were purchased from Animal Facility of Aging Science in Korea Basic Science Institute (Gwangju, Korea). Animals were anesthetized by intramuscular injection of a combination of anesthetics (80 mg/kg ketamine and 12 mg/kg xylazine) and sacrificed. All animal experiments were approved and performed following the rules of Institutional Animal Use and Care Committee of the Chonnam National University. All experimental protocols were carried out in accordance with the relevant guidelines, including any relevant details.

### Q-PCR analysis

Total RNA was isolated from flow cytometry sorted cells or small intestine using the TRIzol reagent (Invitrogen), according to the manufacturer’s instructions. mRNAs were reverse transcribed to cDNAs with a RT PreMix Kit (Enzynomics) and then analyzed by the real-time PCR system (Qiagen) using SYBR Green PCR Master Mix (Enzynomics). All data were normalized to GAPDH expression. Primer sets used are shown in Supplementary Table 1.

### Preparation of resident macrophages in LP

Resident macrophages in LP were isolated by collagenase dissociation method as described previously^9^. The entire intestine of the mice at different ages was cut into 3–4 pieces and then inverted on polyethylene tubes (Becton Dickinson), washed three times with calcium- and magnesium-free phosphate buffered saline (PBS; Lonza) and mucus was removed with 1 mM dithiothreitol (DTT; Sigma-Aldrich). The intestinal epithelium was eluted with 30 mM EDTA at room temperature, followed by digestion with 36 U/ml type IV collagenase (Sigma-Aldrich) in Dulbecco’s Modified Eagle’s Medium (DMEM; Lonza) containing 5% fetal bovine serum (FBS) for 90 minutes at 37 °C in a 5% CO^2^ humidified incubator. The digested tissue was gently shaken for 10 minutes at room temperature and then passed through a 70 μm nylon cell strainer and washed with DMEM.

To purify CD11b and F4/80 expressing macrophages, isolated cells were incubated in 10% serum and Fc block, 1:100 (BD Pharmingen) for 20 minutes at 4 °C and stained with FITC-conjugated anti-CD11c, 1:100 (HL3; eBiosciences), PE/Cy7 conjugated anti-F4/80, 1:100 (BM8; eBiosciences), APC eFluor^®^780 conjugated anti-MHCII (I-A/I-E), 1:100 (M5/114.15.2; eBiosciences), PE conjugated anti-CD11b, 1:100 (M1–70; eBiosciences), and APC conjugated anti-CD206, 1:100 (C068C2; BioLegend), and enriched with a BD FACS Aria II sorter (BD Bioscience) and then analyzed by FlowJo 7.2.2 software (TreeStar).

### Immunofluorescent staining Analysis

Small intestine was collected, opened by longitudinal incision, and fixed with formalin after PBS washing. After blocking with 5% donkey serum in PBS-T (0.3% Triton X-100 in PBS) for 1 hr at room temperature, the tissue samples were incubated with one or more of the following primary antibodies: hamster anti-mouse PECAM-1 antibody, hamster clone 2H8, dilution ratio 1:200 (Chemicon International); rat anti-mouse VE-cadherin antibody, 1:200 (BD Biosciences); Rabbit anti-mouse NG2 antibody, 1:200 (Abcam); Rabbit anti-mouse angiopoietin 2 antibody, 1:100 (Abcam); Rat anti-mouse PDGFR-β antibody, 1:100 (BD biosciences). For visualization, the samples were stained with the following secondary antibodies: FITC-conjugated anti-hamster IgG antibody, 1: 200 (Jackson Immuno Research); Alexa 568-conjugated goat anti-rat antibody, 1:100 (Invitrogen); Alexa 568-conjugated goat anti-rabbit antibody, 1:100 (Invitrogen). Fluorescent signals from whole mounted tissue were imaged with a Zeiss Apotome microscope and a Zeiss LSM 510 confocal microscope equipped with argon and helium-neon lasers (Carl Zeiss). Co-localization density was measured with LSM image browser software (Carl Zeiss).

### Vascular leakage assay

To visualize and determine the vascular leakage, 70-kDa TRITC–dextran (Invitrogen) was used as tracer^14^. The animal was intravenously injected with TRITC-dextrans (2 mg/ 20 g of body weight). Thirty min after injection, the mice were anesthetized and transcardial perfusion was performed with 10 ml of saline to remove remaining tracer from vessels. The small intestine was collected and fixed with 10% neutralized formalin. Confocal microscope image was acquired after stain of vascular network with anti-CD31 antibody.

To quantify the vascular leakage in small intestine, mice were treated as described above and small intestine was homogenized using liquid nitrogen, resuspended in TBS containing 0.1% Triton X-100. After centrifugation, supernatant was analyzed using Fluorometer (TECAN) with excitation/emission wavelengths of 544/590 nm.

### Assessment of ANG2 expression levels in HUVEC

HUVEC grown in EBM2 media were treated with TNF-α (Sigma Aldrich, 10 ng/ml) for 10 hours. Alternatively, HUVEC grown in EBM2 media were co-cultured with macrophages isolated from mouse gut at young and old ages in trans-well plates (Corning, 3 nm/pore) for indicated time. ANG2 expression in the HUVEC was determined by Q-PCR.

### Statistical analysis

Data were analyzed using Prism statistical software. The data met assumptions of a normal distribution as determined by statistical software, and variance was estimated with s.e.m. The two-tailed Student’s t-test was used for statistical analysis between two groups. Differences were considered statistically significant at p < 0.05.

## Additional Information

**How to cite this article**: Jeong, J.-H. *et al*. Microvasculature remodeling in the mouse lower gut during inflammaging. *Sci. Rep.* **7**, 39848; doi: 10.1038/srep39848 (2017).

**Publisher's note:** Springer Nature remains neutral with regard to jurisdictional claims in published maps and institutional affiliations.

## Supplementary Material

Supplementary Information

Supplementary Video 1

Supplementary Video 2

Supplementary Video 3

Supplementary Video 4

Supplementary Video 5
