# Supplementary material for: Microvasculature remodeling in the mouse lower gut during inflammaging
Source: Sci Rep. 2017 Jan 3;7:39848. doi: 10.1038/srep39848 (PMC5206655; doi:10.1038/srep39848)
Supplement: Supplementary Information [file srep39848-s1.pdf]

Microvasculature remodeling in the mouse lower gut during inflammaging

Jae-Ho Jeong[1,2*], KwangSoo Kim[1,2*], Daejin Lim[1,2], Kun-Hee Kim[1,2], Hyung-Seok Kim[3], Sungsu Lee[4], Joo-Hye Song[5], Byoung-Gon Moon[5], Hyon E. Choy[1,2,7], Sang Chul Park[5,6,7]

SUPPLEMENTARY INFORMATION

Figure S1

A

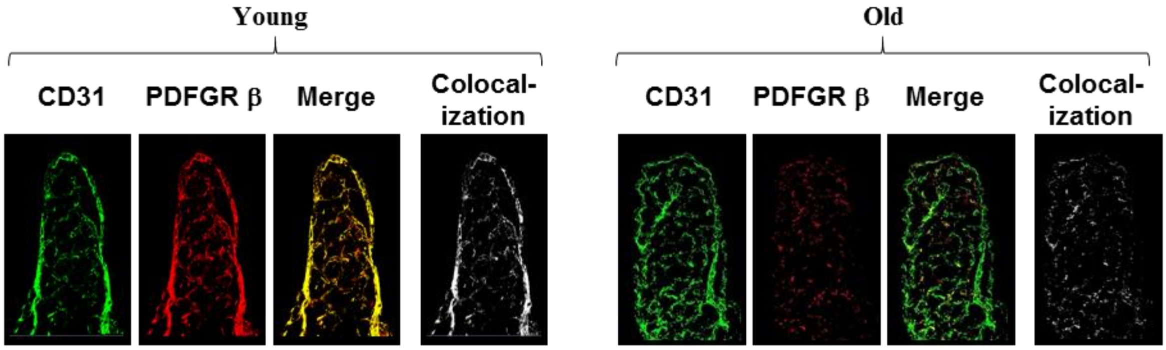

B

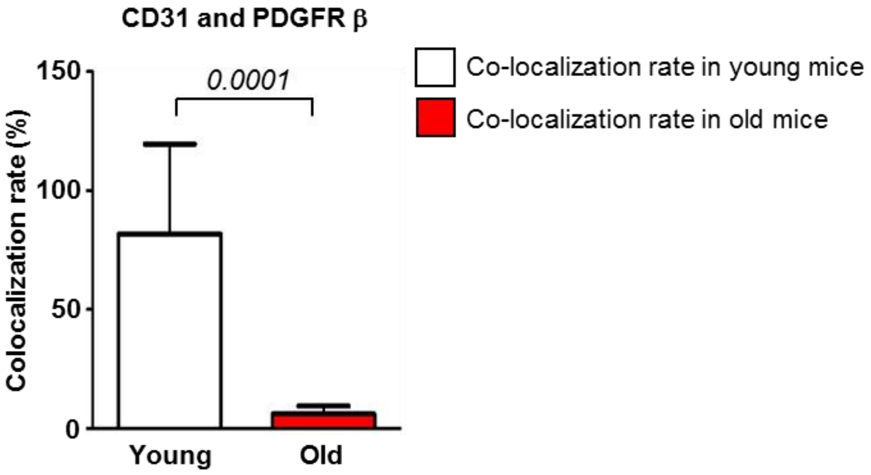

Supplementary Figure 1.

Microvasculatures in LP of young and old mouse lower gut. Presence of pericytes in young and old mice were shown by a whole mount confocal microscopic IF image of LP using specific antibody against PDGFR-β (400 x magnification) (n=10). CD31 signals represent vascular endothelial cells. B. Quantification of co-localization index between CD31 and PDFGR -β in young and old mice gut.

Figure S2

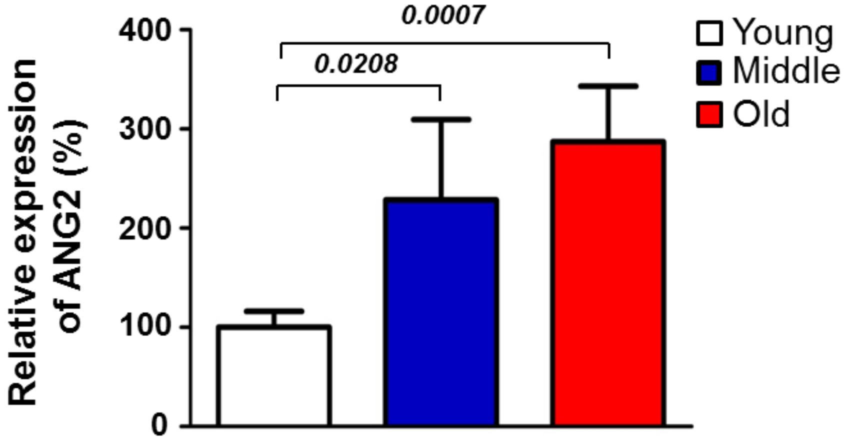

Supplementary Figure 2. Expression levels of ANG-2 from intestinal lysate as assessed by Q-PCR

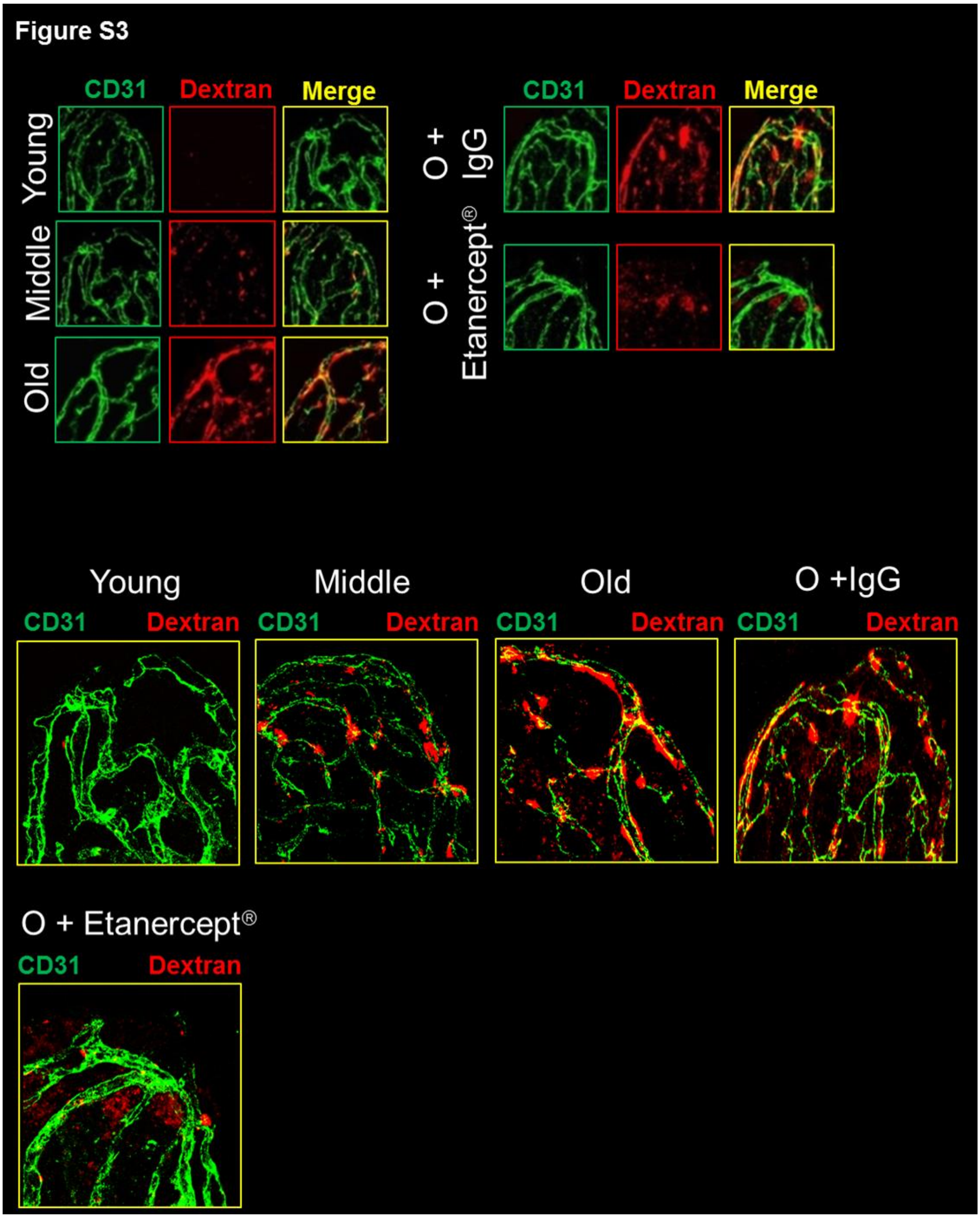

Supplementary Figure 3.

Top panels show high magnification (x100) of images at villi tip shown in Fig. 1D. Bottom panels show extravascular TRITC-dextran shown in Fig. 1D reconstructed in 3-dimensional image using LSM image browser software (Carl Zeiss).

Figure S4

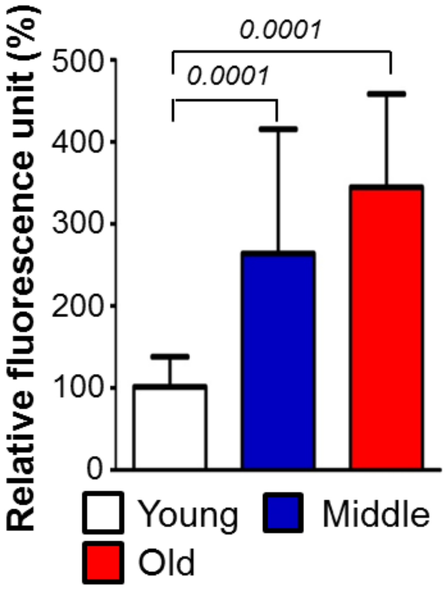

Supplementary Figure 4. Animals (n=18) were treated as described in Fig. 1D legend. Entire small intestine was homogenized using liquid nitrogen, resuspened in TBS-T, and the extravascular TRITC-dextran was quantified using a flourometer.

Figure S5

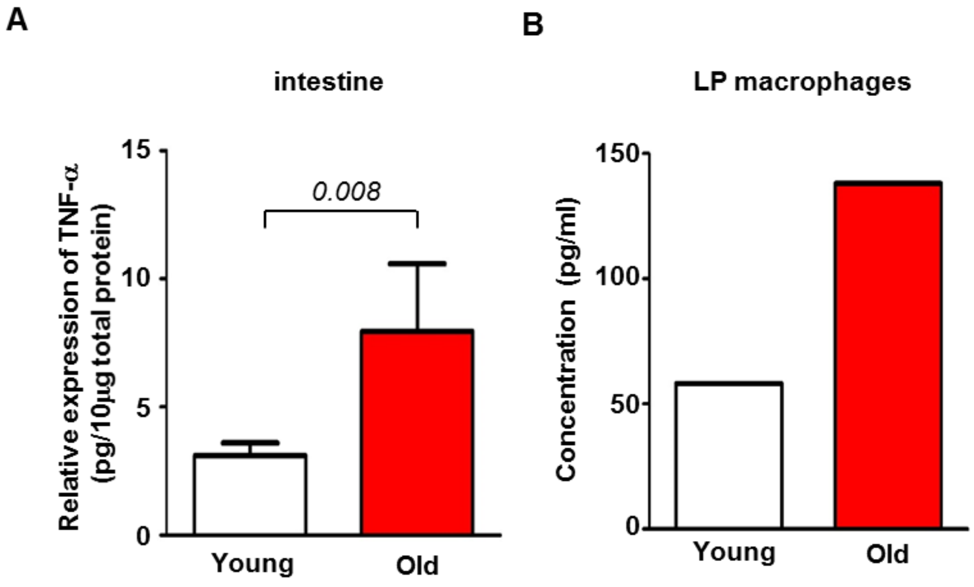

Supplementary Figure 5.

The protein level of TNF-α was determined in intestinal lysate (A) and macrophages (B) from LP of young and old mice (n=5). TNF-α were measured by sandwich enzyme-linked immunosorbent assay (ELISA, eBioscience, San Diego, California, USA). The small intestines were extracted, homogenized in RIPA buffer containing protease inhibitors (0.1 mmol/L phenylmethylsulfonyl fluoride, 0.1 mmol/L benzamidine chloride, 10 µg/ml aprotinin A, and 100 µg/ml leupeptin), and TNF-α in the supernatant was determined (A). Intestinal macrophages (F4/80^{+}, MHCII^{+}) were isolated by FACS sorter and were grown in DMEM media for 18 hrs. TNF-α in the cultured media was determined (B).

Supplementary Table 1. Primer sequences used for quantitative RT-PCR

Primer	Forward (5`→3`)	Reverse (5`→ 3`)
Mouse CCL2	GCTGGAGAGCTACAAGAGGATCA	TCTCTCTTGAGCTTGGTGACAAAA
Mouse Arginase	ATGCAAGAGACCTTCAGCTAC	GCTGCTTTCCCAAGAGTTGGG
Mouse IL-6	AGGATACCACTCCCAACAGACCT	CAAGTGCATCATCGTTGTTCATAC
Mouse IL-10	TGGCCCAGAAATCAAGGAGC	CAGCAGACTCAATACACACT
Mouse TNFα	TTCTGTCTACTGAACTTCGGGGTGATCGGTCC	GTATGAGATAGCAAATCGGCTGACGGTGTGGG
Mouse VEGF-A	GGAGATCCTTCGAGGAGCACTT	GGCGATTTAGCAGATATAAGAA
Mouse ANG2	GCATGACCTAATGGAGACCGTC	GATAGCAACCGAGCTCTTGGAG
Mouse IL-1β	CCAGCTTCAAATCTCACAGCAG	CTGATGAGAGCATCCAGCTTCA
Mouse GAPDH	ACCACAGTCCATGCCATCAC	CACCACCCTGTTGCTGTAGCC
Human ANG2	TCCAAGCAAAATTCCATCATTG	GCCTCCTCCAGCTTCCATGT
Human GAPDH	AGGGCTGCTTTTAACTCTGGT	CCCCACTTGATTTTGGAGGGA

Supplement information of Video files

Video 1

3-dimensional image of ‘Young’ in supplementary figure3 (bottom panels)

Video 2

3-dimensional image of ‘Middle’ in supplementary figure3 (bottom panels)

Video 3

3-dimensional image of ‘Old’ in supplementary figure3 (bottom panels)

Video 4

3-dimensional image of ‘O+IgG’ in supplementary figure3 (bottom panels)

Video 5

3-dimensional image of ‘O+Etanercept’ in supplementary figure3 (bottom panels)
